# The association of circulating miR-191 and miR-375 expression levels with markers of insulin resistance in overweight children: an exploratory analysis of the I.Family Study

**DOI:** 10.1186/s12263-021-00689-1

**Published:** 2021-07-09

**Authors:** Giuseppe Iacomino, Fabio Lauria, Paola Russo, Antonella Venezia, Nunzia Iannaccone, Pasquale Marena, Wolfgang Ahrens, Stefaan De Henauw, Dénes Molnár, Gabriele Eiben, Ronja Foraita, Antje Hebestreit, Giannis Kourides, Luis A. Moreno, Toomas Veidebaum, Alfonso Siani, on behalf of the I.Family Consortium

**Affiliations:** 1 Institute of Food Sciences, National Research Council, ISA-CNR, via Roma 64, 83100 Avellino, Italy; 2 Leibniz Institute for Prevention Research and Epidemiology, BIPS, Achterstraße 30, 28359 Bremen, Germany; 3 University of Ghent, C. Heymanslaan 10, 4K3, 9000 Ghent, Belgium; 4 Department of Pediatrics, Medical School, University of Pécs, Pécs, Hungary; 5 Sahlgrenska Academy at the University of Gothenburg, Medicinaregatan 3, 413 90 Göteborg, Sweden; 6 Research and Education Institute of Child Health, ave, #205 2015, Strovolos, 138 Limassol, Cyprus; 7 University of Zaragoza, Domingo Miral, s/n, 50009 Zaragoza, Spain; 8 National Institute for Health Development, Hiiu 42, 11619 Tallinn, Estonia

**Keywords:** MicroRNAs, Insulin impairment, Overweight/obese, Children/adolescents, Biomarkers

## Abstract

**Background:**

In recent years, the exciting emergence of circulating miRNAs as stable, reproducible, and consistent among individuals has opened a promising research opportunity for the detection of non-invasive biomarkers. A firm connection has been established between circulating miRNAs and glycaemic as well as metabolic homeostasis, showing that levels of specific miRNAs vary under different physio-pathological conditions.

**Objective:**

In this pilot study, we investigated the expression of candidate miRNAs, hsa-miR-191-3p and hsa-miR-375, in relation to biomarkers associated with insulin sensitivity in a subgroup (n=58) of subjects participating to the European I.Family Study, a project aimed to assess the determinants of eating behaviour in children and adolescents and related health outcomes. The sample included overweight/obese children/adolescents since overweight/obesity is a known risk factor for impaired glucose homeostasis and metabolic disorders. Biological targets of candidate miRNAs were also explored in silico.

**Results:**

We observed a significant association of the two miRNAs and early changes in glycaemic homeostasis, independent of covariates including country of origin, age, BMI z-score, puberty status, highest educational level of parents, total energy intake, energy from fats, energy from carbohydrates, and energy from proteins.

**Conclusion:**

Identification of circulating miRNAs associated with insulin impairment may offer novel approaches of assessing early variations in insulin sensitivity and provide evidence about the molecular mechanisms connected to early changes in glycaemic homeostasis.

**Trial registration:**

ISRCTN, ISRCTN62310987. Retrospectively registered, http://isrctn.com/ISRCTN62310987

**Supplementary Information:**

The online version contains supplementary material available at 10.1186/s12263-021-00689-1.

## Highlights

- Circulating miR-191-3p and miR-375 are connected to early changes in glycaemic homeostasis in healthy overweight/obese children and adolescents.
- Circulating miRNAs associated with insulin impairment may offer novel methods of assessing early variations in insulin sensitivity.
- Identification of circulating miRNAs connected with insulin resistance would have the potential to offer innovative methods of assessing insulin sensitivity to detect prediabetes and provide further evidence about the underlying molecular mechanisms connected to insulin impairment.

## Background

Insulin resistance is a pathological condition in which the body’s cells become resistant to the effects of insulin [1]. This condition is strictly interconnected to the disruption of the hormone-signalling pathway, which may include either defects in pancreatic insulin secretion or failure of target cells to uptake and metabolize glucose in response to insulin, or both conditions [2]. Taken together, the high levels of glucose, insulin, and IGF-1, which result from insulin resistance, may be connected to the onset of obesity, metabolic syndrome, cardiovascular diseases, and others chronic diseases, by unsettling basic metabolic processes [3]. Besides, dysregulation in insulin signalling is among the typical and earliest metabolic signs predisposing to the development of type 2 diabetes (T2D) [4, 5]. This latter condition is a less defined disorder since clinical features of T2D are not commonly perceived until pancreatic islets start to fail, and clinical diagnosis is delayed until systemic complications start and irreversible damage has already occurred. Although key mechanisms leading to insulin resistance are not completely understood, both genetic and environmental factors seem to be involved [6]. Currently, T2D represents a major health concern since its prevalence has been dramatically increased for the last decade [7], accounting for 90–95% of total diabetes worldwide, not only in adults but also in children [8].

In recent years, numerous efforts have been made to identify early, reliable, and predictive biomarkers of altered insulin sensitivity either in individuals or case-control studies (i.e. obese). Nevertheless, risk-screening methods are nearly limited to the detection of unhealthy metabolic conditions, including static or dynamic detection of the levels of insulin, glucose, and the interrelated increase in circulating protein glycation [9]. Each of these methods has limitations, and new studies, finalized to a better understanding of the physio-pathological basis of this condition, are straightaway required to improve new diagnostic and prognostic opportunities [10].

Emerging evidence suggests an association between microRNAs (miRNAs) and diseases [11]. miRNAs have recently emerged as peacekeepers of body homeostasis, playing pivotal roles in the physiopathology of many processes, including body energy balance and metabolic homeostasis [12], and several studies have established that miRNAs correlate in a causative manner with obesity, metabolic syndrome, T2D, and other non-communicable diseases [13, 14]. miRNAs are components of epigenetic mechanisms modulating the expression of messenger RNAs (mRNAs): at present, up to 2599 different miRNAs have been identified in humans (miRTarBase, release 8.0) [15]. This class of small RNAs acts as post-transcriptional regulators of gene expression by base-pairing with their target mRNAs. Each miRNA can target many mRNAs, and an individual transcript may include several miRNAs binding sites. miRNAs can also be packaged and released from cells arranged either into exosomes or protein complexes [16]. The discovery of about 300 circulating miRNAs in plasma and other body fluids has highlighted their potential as key intercellular signalling molecules and disease biomarkers [17]. Accordingly, dysregulation of miRNAs has been shown to reflect the status and functions of different tissues and organs, probably contributing to their abnormalities [18].

A pivotal role for miRNAs in controlling glycaemic homeostasis was first established in 2004 by Poy et al. who showed that miR-375 is involved in the regulation of insulin secretion in pancreatic *β*-cells [19]. More recently, several additional miRNAs have been identified as key epigenetic elements contributing to glucose homeostasis in both T1D and T2D conditions [20–25]. As far as we know, few studies have focused on early variations in insulin sensitivity in young people [26] due to the difficulties in distinguishing pre-diabetes conditions in general healthy populations for variabilities in clinical parameters and the effect of lifestyle factors [27]. Distinctive miRNA signatures could be of help to predict early conditions potentially involved in the development of T2D, as well as to check the efficacy of pharmacological and nutritional interventions [27]. In a previous paper [28], aimed to evaluate the association of circulating miRNAs with obesity in a sample of normal weight (NW) and overweight/obese (OW/Ob) children, we found that hsa-miR-191-3p was associated with obesity and also with markers of insulin sensitivity.

The aim of the present study is to confirm this association and, possibly, to identify other miRNAs potentially linked with early glycaemic impairment in a subsample of only OW/Ob children, participating in the I.Family Study [29], since obesity has a causal influence on glucose homeostasis and diabetes onset [30].

## Results

### Anthropometric and metabolic characteristics of the study sample

The anthropometric and metabolic characteristics of the participants are summarized in Table 1. The mean age was 12.2 for girls and 12.4 for boys. There were no obvious differences regarding the characteristics between males and females. However, insulin levels and HOMA index were slightly higher in girls, although the difference did not reach statistical significance.

**Table 1:** Characteristics of overweight/obese subjects included in the study

		Boys (27)	Girls (31)	*p*
Age	(years)	12.4 ± 1.8	12.2 ± 1.6	0.816
Puberty	(% yes)	68.0	54.8	0.316*
BMI z-score		2.0 ± 0.5	2.0 ± 0.6	0.483
TC	(mg/dl)	153.5± 21.7	155.8 ± 21.9	0.692
TRG	(mg/dl)	80.04 ± 36.2	79.8 ± 45.0	0.985
HDL	(mg/dl)	53.0 ± 11.0	51.6 ± 11.7	0.629
LDL	(mg/dl)	88.2 ± 21.0	91.8 ± 19.4	0.501
Glu	(mg/dl)	93.7± 7.4	93.9 ± 6.8	0.899
HBA1c	(%)	4.9 ± 0.2	5.0 ± 0.3	0.314
Insulin	(pg/dl)	379.0 ±215.0	476.5.8 ± 383.8	0.308
HOMA index		2.2 ± 2.0	2.7 ± 2.2	0.353
Total energy intake	(kcal/day)	1703± 577	1719 ± 657	0.925
Energy from fat	(%)	29.3 ± 10.3	26.2 ± 7.1	0.176
Energy from CHO	(%)	55.2 ± 11.5	59.0 ± 10.2	0.181
Energy from protein	(%)	14.3 ± 4.2	15.4 ± 4.8	0.400
miR-191-3p	(relative level)	3.8 ± 5.4	3.3 ± 1.5	0.623
miR-375	(relative level)	101.8 ± 314.7	150.8 ± 370.9	0.593

Data are expressed as mean ± SD or as frequency (%)

*Glu* blood glucose, *LDL* low-density lipoprotein, *HDL* high-density lipoprotein, *HOMA* index homeostasis model assessment of insulin resistance, *HBA1* haemoglobin A1c, *CHO* carbohydrate, *TRG* triglycerides, *TC* total cholesterol

*Chi-square

### RT-qPCR validation in individual plasma samples

Designated miRNAs were evaluated in individual samples by RT-qPCR using a cohort of 58 subjects. Following extraction of single samples, miRNA relative levels were normalized using the spike-in Cel-miR-39. Differences in miRNA signature with respect to anthropometric and biochemical variables were analysed. Noteworthy, increased levels of serum insulin are typically detected in subjects with pre-diabetes and T2D [31]. In the whole study sample (Table 2), the association of the candidate de-regulated miRNAs, hsa-miR-191-3p and hsa-miR-375, with both insulin level and HOMA index was confirmed in OW/Ob children. Given sex differences in insulin levels and susceptibility to develop insulin resistance [32], we further considered boys and girls separately. As shown in Table 3, the differential expression of candidate miRNAs was statistically confirmed to be associated with insulin level and HOMA index in both girls and boys.

**Table 2:** Association between miRNAs and insulin sensitivity in the whole study sample (n= 58)

	miR-191 Coefficient (95% CI)	***p***	miR-375 Coefficient (95% CI)	***p***
**Insulin (pg/dl)**	457.3 (369.7–545.0)	**<0.001**	454.8 (375.9–533.7)	**<0.001**
**HOMA index**	2.6 (2.0–3.1)	**<0.001**	2.5 (2.0–3.0)	**<0.001**

*95% CI* 95% confidence interval. Covariates: sex, country of origin, highest educational level of parents defined according to the International Standard Classification of Education (ISCED, 2011), puberty status, age, total energy intake (kcal/day), energy from fat (%), energy from CHO (%), energy from protein (%). Data are expressed as mean (95% confident intervals)

**Table 3:** Association between miRNAs and insulin sensitivity in boys and girls

	Boys (*n* = 27) Coefficient (95% CI)	*p*	Girls (*n* = 31) Coefficient (95% CI)	*p*
A) miR-191-3p				
Insulin (pg/dl)	413.2 (309.8–516.6)	**0.004**	520.3 (436.0–604.6)	**0.007**
HOMA index	2.3 (1.4–3.2)	**0.010**	2.9 (1.8–3.4)	**0.003**
B) miR-375				
Insulin (pg/dl)	444.2 (346,6–541.8)	**0.003**	459.5 (370.6–548.4)	**0.009**
HOMA index	2.5 (1.6–3.4)	**0.009**	2.6 (1.9–3.2)	**0.034**

*95% CI* 95% confidence interval. Covariates: country of origin, highest educational level of parents defined according to the International Standard Classification of Education (ISCED, 2011), puberty status, age, total energy intake (kcal/day), energy from fat (%), energy from CHO (%), energy from protein (%). Data are expressed as mean (95% confident intervals)

### Bioinformatics

To gain a mechanistic understanding of how the miRNAs could be associated with glycaemic impairment, molecular interactions of confirmed miRNAs were predicted by bioinformatics.

The functional characterization and enrichment of the biological pathways potentially modulated by the two miRNAs were investigated by miRPath analysis in which the two miRNAs were combined. Target genes were classified according to KEGG functional annotations to identify the key pathways regulated by miRNAs. Identified pathways were arranged according to enrichment statistical scores (*p*-values) in addition to the number and names of miRNA target genes implicated in each KEGG pathway. Expected pathways were determined either for single miRNAs or in their association since both donor and target tissues of miRNAs are undisclosed. Computational predictions designated the role of candidate miRNAs in controlling the expression of genes involved in relevant biological processes with several genes associated with control of glucose metabolism (Table 4).

**Table 4:** KEGG pathways of differentially expressed miRNAs in early glycaemic impairment

	*p*	n. of genes	n. of miRNAs
A) KEGG pathway			
Huntington’s disease	0.000000019	3	1
Hippo signalling pathway	0.0000013	18	2
Lysine degradation	0.000046	8	1
Protein processing in endoplasmic reticulum	0.000239	25	1
Proteoglycans in cancer	0.002373	22	1
Viral carcinogenesis	0.004420	26	1
Glycosaminoglycan biosynthesis - chondroitin sulfate/dermatan sulfate	0.007630	2	1
Colorectal cancer	0.013249	3	1
Central carbon metabolism in cancer	0.023230	9	1
Endocytosis	0.026468	22	1
Transcriptional misregulation in cancer	0.027697	4	1
Thyroid hormone signalling pathway	0.038555	13	1
FoxO signalling pathway	0.041500	17	1
Adherens junction	0.041500	10	1
B) KEGG pathway			
Hippo signalling pathway	0.00000139	18	2

A) Pathways enrichment analysis of single deregulated miRNAs. B) Pathways union of miRNAs is also reported. Pathways were classified according to KEGG functional annotations to identify top pathways that were actively regulated by miRNAs. Merged *p*-value is extracted by combining calculated significance levels using Fisher’s exact test (hypergeometric distribution) with a *p*-value threshold=0.05 and MicroT threshold=0.8

Attractively, the screening of the tissue expression pattern of the dysregulated miRNAs over the tissue atlas IMOTA established that these miRNAs are differentially expressed in several tissues (Supplementary Fig.1).

## Discussion

The discovery of the molecular basis for insulin resistance along with glycaemic impairments has emerged as one of the greatest challenges of modern medicine. Although the underlying mechanism responsible for insulin resistance is still uncertain, deficiencies in insulin signalling are considered either driving factors or primary signs predisposing to the development of T2D. There is consequently a need to improve approaches for early detection, to allow intervention strategies to be significantly more effective.

In previous papers, we showed that characteristic circulating miRNA profiles are associated with childhood obesity in a sample of NW *vs* OW/Ob [28, 29]. In the present analysis, we investigated the expression of two candidate miRNAs, hsa-miR-191-3p and hsa-miR-375, in relation to biochemical parameters associated with early impairments in insulin sensitivity in a subgroup of only OW/Ob European children/adolescents, since obesity has a pivotal influence on glucose homeostasis and primary metabolic damage—the term “diabesity” has been coined in recent times [30]. In our analyses, the study sample was stratified by sex, since it is well known that the effects of insulin, the susceptibility to develop insulin resistance, and the response to stimuli that notoriously modulate the effects of insulin, body composition, and energy balance are all sex-related characteristics [32, 33].

In our screening, the association of the two miRNAs with the markers of early glycaemic impairment was first observed in the whole study sample and then confirmed in girls and boys separately. The analyses were adjusted for confounding factors, including puberty since puberty is characteristically associated with a decrease in insulin sensitivity [34]. Covariates also included the highest educational level of parents since several studies indicated that parental influences have a marked effect on nutritional habits, dietary intakes, and food preferences of young children [35]. Of note, no one of the study-subject suffered from metabolic syndrome or TD2; no alterations in blood levels of glucose and of HbA1c were recognized.

Pancreatic failure is a common characteristic of different forms of diabetes. Many miRNAs have been previously described to be involved in pancreatic development and β-cell activity, with some of them having positive roles, while others exercising negative roles [36, 37]. One of the most relevant, miR-375 was identified as predominant in pancreatic islets and critical in preserving pancreatic β-cell mass and regulating insulin secretion [38]. An increase in miR-375 was found during islet development, whereas β-cells’ activity is coupled to its decline [39]. Moreover, miR-375 targets several transcription factors as PDX1, HNF6, and INSM1, engaged in pancreatic islets activity [40, 41]. Inhibition of miR-375 in animal model prompts major defects in islet development [15]. Yet, inhibition of miR-375 induces improved insulin secretion, while miR-375 overexpression attenuates insulin release by targeting myotrophin, a protein involved in insulin granule fusion, by inhibiting exocytosis [19]. Concurrently, it has been shown that miR-375 downregulates insulin expression by targeting the phosphoinositide-dependent kinase-1 [42]. Interestingly, Sedgeman et al. also found that pancreatic β-cells are engaged in exporting miR-375 to HDL and that this process is inversely regulated to insulin secretion [43]. Furthermore, miR-375 has been shown to control blood glucose homeostasis and induce adipogenic differentiation, both processes associated with T2D [44]. High levels of miR-375 are also established in the islet of T2D patients in comparison with healthy subjects and an animal model of obesity and insulin resistance. Similarly, human blood-based higher expression of miR-375 was detected in T2D patients [45]. Several reports suggest the potential involvement of epigenetic mechanisms in developing T2D as a crucial boundary between the effects of genetic predisposition and environmental effects [46]. Unfortunately, scientific evidence is limited emphasizing the need for further investigations to establish whether epigenetic marks may affect the risk of T2D [46]. Of note, Yin et al. evaluated the methylation pattern at multiple CpG units within the promoter regions of miR-375 from the Chinese Han T2D subjects [47]. Authors found statistically significant differences in hyper-methylation in one specific CpG units in the T2D subjects compared with the control group; concurrently, the transcription level of mature miR-375 was significantly upregulated in the T2D compared with matched control group. Inclusively, the study suggested that the aberrant hyper-methylation of miR-375 has a role in the regulation of miR-375 levels and could be added to the known risk factors to predict T2D progression.

miR-191 is expressed as part of the miR-191/425 cluster which is highly conserved in metazoan species. miR-191 has been found dysregulated in a large number of different types of human cancers. Early studies identified miR-191 as an oestrogen-inducible miRNA in an ER-dependent manner and acting as a critical mediator of oestrogen-mediated cell proliferation [48]. Interestingly, previous researches also confirmed an abnormal expression of miR-191 in diabetes. As an example, a study showed that miR-191 is downregulated in adult peripheral T-*reg* cells of T1D compared with healthy controls [49]. This miRNA represents a key player in aetiology of diseases since relevant cellular processes such as cell proliferation, differentiation, and apoptosis are controlled, with TGF-β and MAPK pathways found to be most regulated by this miRNA [50]. Our results are also in line with an epidemiological study demonstrating a positive association between miR-191 and glycaemic impairment/progression in adult Asian Indians, an ethnic group that notoriously has a high incidence of T2D [51].

Further studies have shown the role of miRNAs as attractive potential biomarkers for obesity and associated risk factor diseases. In this context, Jimenez-Lucena et al. found that circulating miRNA levels combined with HbA1c could be useful in predicting T2D development in adults [52]. As well, miR-126 has been demonstrated to be a useful marker for metabolic diseases in children [53]. Several research has proposed that additional circulating miRNAs are associated with T2D including miR-15a [52], miR-126 [54], miR-146a [55], miR-375 [56] among the others. miR-491-5p, miR-1307-3p, and miR-298, have also been recognized, as predictive tools of T2D in prediabetes patients [57, 58]. In a recent study, Parrizas et al. provided evidence for the existence of a circulating miRNA signature for prediabetes by identifying two different miRNAs, miR-10b and miR-223-3p, whose combination was able to differentiate between progressor and non-progressor prediabetic subjects at a stage at which other features, including glycaemia, are less useful in separating them [59]. That study was conducted on a subset of extracellular miRNAs enclosed inside microvesicles and highlights the ability of miRNA screening as a useful tool for adult subjects at risk of developing diabetes. In our research, based on plasma circulating miRNAs, we focused on the association between differentially expressed miRNAs and biochemical proxies of insulin resistance in children.

## Conclusions

In conclusion, in this exploratory study, we attempted to shed light on the role of circulating miRNAs in early changes in glycaemic homeostasis associated with childhood obesity. However, confirmatory studies are required given the small sample sizes and the lack of long-term outcome data. Besides, further functional studies are expected to identify and validate the molecular targets of the miRNAs differentially expressed in early incident glycemic impairment.

Identification of circulating miRNAs connected with insulin resistance would have the potential to offer innovative methods of assessing insulin sensitivity to detect prediabetes and provide further evidence about the underlying molecular mechanisms connected to insulin impairment. Finally, the discovery of early predictors would considerably diminish the social and economic costs currently associated with T2D, that is habitually undetected until hyperglycemia and its complications are identified.

## Methods

### Study sample

This study was conducted on a subgroup of healthy OW/Ob European children/adolescents (n=58) of the I.Family Study, an EC-funded project aiming at investigating determinants of food choice, lifestyle, and related health outcomes in children and adolescents of eight European countries (Belgium, Cyprus, Estonia, Germany, Hungary, Italy, Spain, and Sweden). A complete description of the project (registration number ISRCTN62310987) has been published earlier [60]. The research was conducted according to the standards of the Declaration of Helsinki. Approval by the national ethics committees was obtained by each of the participating centres carrying out the fieldwork. Anthropometric and dietary intake data were collected using standardized procedures and a detailed description of methods has been reported elsewhere [61]. A complete description of the subsample and the selection criteria can be found in Iacomino et al. [28]. Briefly, in each country, we first selected 20 children who retained overweight or obesity, i.e. who had a BMI z-score of more than + 1 at baseline and after 2 years at follow-up, respectively, and did not change more than ± 0.1 in BMI z-score per year (defined as overweight/obese) [62]. In the present, the analysis was conducted in a subsample of 58 OW/Ob children/adolescents of the I.Family Study (Belgium n=4, Cyprus n=4, Estonia n=10, Germany n=11, Hungary n=7, Italy n=9, Spain n=4, and Sweden n=9), with a complete dataset for the variables of interest. Information about educational level of parents and pubertal status were collected using a questionnaire filled in at home by parents [63].

### Biochemical analysis

The fasting venous blood was collected in BD Vacutainer® blood collection tubes according to standard operating procedures. A comprehensive description of sample collection and analytical procedures has been previously published [64]. Glucose, glycated haemoglobin (HbA1c), and serum insulin levels were measured as part of routine laboratory testing, in a central laboratory (Laboratoriumsmedizin Dortmund Dr Eberhard und Partner GbR). Insulin resistance was estimated by the homeostatic model assessment (HOMA) index calculated according to the formula: HOMA = serum insulin (mU/l) × blood glucose (mg/dl)/405.

### miRNA profiling

Taking advantage of the qPCR array technology, we previously confirmed that altered circulating miRNA profiles are associated with childhood obesity in I.Family Study subjects [28]. In that study, we described circulating miRNAs, potentially associated with glycaemic impairment (data not shown). In the current investigation, we aimed to confirm the association of hsa-miR-191-3p (MIMAT0001618) and hsa-miR-375 (MIMAT0000728) with early changes in insulin sensitivity in a new sample of children/adolescents through validation by SYBR green-based real-time quantitative RT-PCR (RT-qPCR). Experimental methods for miRNA extraction and screening from plasma samples have been earlier established [28, 63]. Briefly, individual plasma samples were screened for haemoglobin levels, haemolysed samples were excluded from the analysis [28]. Individual assays were performed in triplicate by using the miScript Primer Assays, according to the manufacturer’s recommendations (Qiagen, Germany).

### Bioinformatics

Biological targets of miRNAs were in silico explored by using miRPath v3.0 [65] which achieves advanced analysis such as hierarchical clustering of miRNAs and pathways based on the levels of their interactions. miRNA targets were predicted by the DIANA-microT-CDS algorithm or even experimentally validated miRNA interactions derived from DIANA-TarBase v7.0 [65]. A *p*-value threshold of 0.05 and Benjamini Hochberg false discovery rate (FDR) correction were applied to the analysis. Predicted targets were further evaluated through the use of the Kyoto Encyclopedia of Genes and Genomes (KEGG). The distribution of the miRNAs in human tissues was assessed by using IMOTA an Interactive Multi-Omics-Tissue Atlas including a collection of expression profiles from different publicly available miRNA-seq datasets.

### Statistical analysis

Statistical analyses were performed in the whole study sample and stratified by sex using IBM SPSS Statistics (v24.0.; IBM Corp). Data were calculated as means ± standard deviations (SD) or means and 95% confidence intervals (CI), as appropriate. Associations of miRNAs expression with HOMA and insulin was performed using linear regression analyses adjusting for covariates (country of origin, age, BMI z-score, puberty status, highest educational level of parents using the ISCED standard [66], total energy intake, energy from fats, energy from carbohydrates, and energy from proteins). Pearson’s correlation coefficient was used to study associations between the variables. A two-tailed *p*-value less than 0.05 was considered statistically significant.

## Supplementary Information

**Additional file 1: Supplementary Fig. 1.** Tissue expression pattern of the dysregulated miRNAs

## Data Availability

All data generated or analysed during this study are included in this published article.

## Abbreviations

BMI: Body mass index
ER: Estrogen receptor
HOMA: Homeostatic model assessment
IGF: Insulin-like growth factor
miRNA: MicroRNA
NW: Normal weight
OW/Ob: Overweight/obese
T2D: Type 2 diabetes
