# Supplementary figures and images for: The association of circulating miR-191 and miR-375 expression levels with markers of insulin resistance in overweight children: an exploratory analysis of the I.Family Study
Source: Genes Nutr. 2021 Jul 9;16:10. doi: 10.1186/s12263-021-00689-1 (PMC8272322; doi:10.1186/s12263-021-00689-1)

# Supplementary Fig. 1. Tissue expression pattern of the dysregulated miRNAs

## Hsa-miR-375

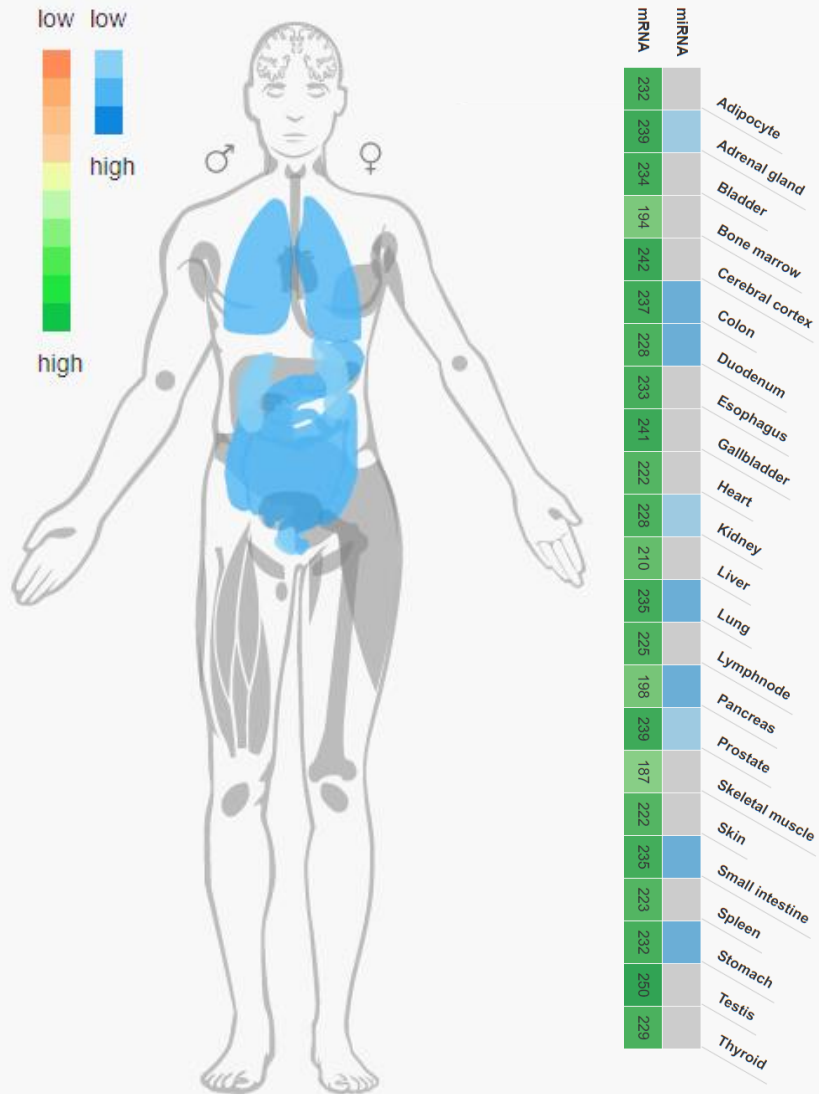

## Hsa-miR-191-3p

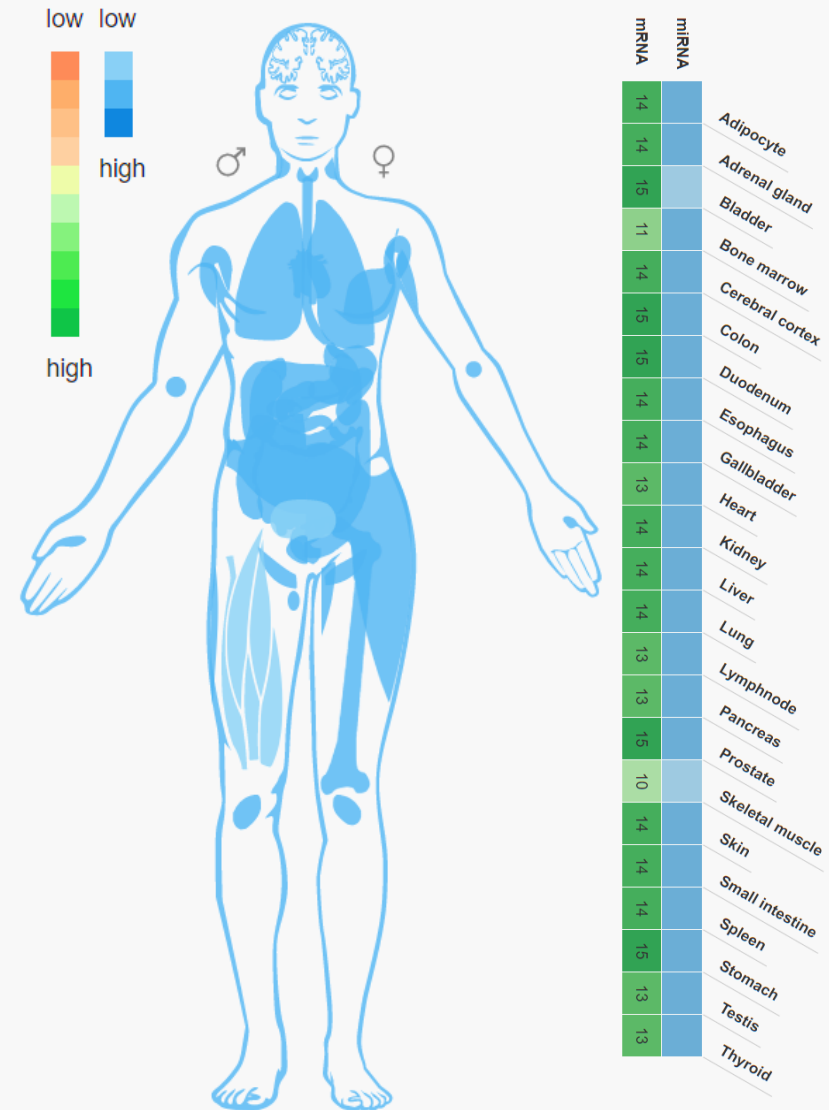

Supplement: Supplementary file 1 — Additional file 1: Supplementary Fig. 1. Tissue expression pattern of the dysregulated miRNAs [file 12263_2021_689_MOESM1_ESM.pdf]
